# Using machine learning of computerized vocal expression to measure blunted vocal affect and alogia

**DOI:** 10.1038/s41537-020-00115-2

**Published:** 2020-09-25

**Authors:** Alex S. Cohen, Christopher R. Cox, Thanh P. Le, Tovah Cowan, Michael D. Masucci, Gregory P. Strauss, Brian Kirkpatrick

**Affiliations:** 1 Department of Psychology, Louisiana State University, Baton Rouge, LA USA; 2 Center for Computation and Technology, Louisiana State University, Baton Rouge, LA 70803 USA; 3 Department of Psychology, University of Georgia, Athens, GA USA; 4 Department of Psychiatry and Behavioral Sciences, University of Nevada, Reno, USA

**Keywords:** Schizophrenia, Biomarkers

## Abstract

Negative symptoms are a transdiagnostic feature of serious mental illness (SMI) that can be potentially “digitally phenotyped” using objective vocal analysis. In prior studies, vocal measures show low convergence with clinical ratings, potentially because analysis has used small, constrained acoustic feature sets. We sought to evaluate (1) whether clinically rated blunted vocal affect (BvA)/alogia could be accurately modelled using machine learning (ML) with a large feature set from two separate tasks (i.e., a 20-s “picture” and a 60-s “free-recall” task), (2) whether “Predicted” BvA/alogia (computed from the ML model) are associated with demographics, diagnosis, psychiatric symptoms, and cognitive/social functioning, and (3) which key vocal features are central to BvA/Alogia ratings. Accuracy was high (>90%) and was improved when computed separately by speaking task. ML scores were associated with poor cognitive performance and social functioning and were higher in patients with schizophrenia versus depression or mania diagnoses. However, the features identified as most predictive of BvA/Alogia were generally not considered critical to their operational definitions. Implications for validating and implementing digital phenotyping to reduce SMI burden are discussed.

## Introduction

Blunted vocal affect (BvA) and alogia, defined in terms of reduced vocal prosody and verbal production, respectively, are diagnostic criteria of schizophrenia^1^ and are present in major depressive, post-traumatic, neurocognitive, and neurodegenerative spectrum disorders^2–4^. BvA and alogia are typically measured using clinical ratings of behavior observed during a clinical interview, and have been associated with a host of functional maladies, such as impoverished quality of life, and poor social, emotional and cognitive functioning^5–7^. Their etiology and biological roots are as yet unknown and treatments alleviating their severity are undeveloped^8^. Given that BvA and alogia reflect overt behaviors that can be quantified, it has long been proposed that computerized acoustic analysis could be used to measure them^9–12^. Presumably, this type of “digital phenotyping”^13^ could be automated to provide a relatively efficient and sensitive “state” measure of negative symptoms; with applications for improving diagnostic accuracy and for efficiently tracking symptom severity, relapse risk, treatment response, and pharmacological side effects^14–16^. Moreover, acoustic analysis is based on speech analysis technologies that are freely available, well-validated for a variety of applications, and can be collected using a wide range of unobtrusive and in situ remote technologies (e.g., smartphones, archived videos)^17^. Despite the existence of several dozen studies evaluating computerized acoustic analysis of natural speech to measure BvA and alogia, there is insufficient psychometric support to consider them appropriate for clinical applications^16^, as they have shown only modest convergence with clinical ratings. For example, in a study of 309 patients with schizophrenia, clinically rated negative symptoms were non-significantly and weakly associated with pause times, intonation, tongue movements, emphasis, and other acoustically derived aspects of natural speech (absolute value of *r*’s, 0.03–0.14)^18^. Null, variable, and even counterintuitive findings are reported in many studies^19–24^. These findings are summarized in meta-analyses of 12 studies^25^ and 55 studies^24^ comparing acoustic features in schizophrenia patients versus controls. Both meta-analyses reported large heterogeneity in effects between studies, and overall effects (with the exception of pause duration mean and variability) that were relatively weak (i.e., range of other *d*’s = −1.18 to 0.33 in ref. ^25^; a range of other *g*’s = −1.26 to −0.05 in ref. ^24^) compared to differences seen with clinical ratings (e.g., *d* = 3.54 in ref. ^25^). While interesting, these findings fall considerably short of the threshold of reliability and validity expected for a clinically deployable assessment tool^18,26,27^. The present study used machine-learning analysis of computerized vocal measures procured from a large sample of patients with serious mental illness (SMI) to redress issues with these prior studies.

The underwhelming/inconsistent convergence between clinical ratings and objective measures of negative symptoms raises questions about whether negative symptoms can be accurately modeled using objective technologies at all. We see two areas for improvement. The first involves context. Like many clinical phenomena, blunted affect and alogia must be considered as a function of their cultural and environmental context. Evaluating alogia, for example, requires a clinician to consider the quantity of speech within the context of a wide array of factors, such as what question was asked, and the patient’s age, gender, culture, and potential motivations for answering. For this reason, a simple word count of spoken words without regard to context may not be informative for quantifying severity of alogia. “Non-alogic” individuals will appropriately provide single word responses in certain contexts, whereas alogic individuals may provide comparatively lengthy responses in different contexts (e.g., a memory test). Most prior studies have failed to systematically consider speaking task, important in that dramatic speech differences in frequency and volume can emerge as a function of social, emotional, and cognitive demands of the speaking task^28–30^. The second area involves the feature set size and comprehensiveness. The human vocal expression can be quantified using thousands of acoustic features based on various aspects of frequency/volume/tone and how they change over time. For example, the winning algorithm of the 2013 INTERSPEECH competition, held to predict vocal emotion using acoustic analysis of an archived corpus, contained ~6500 vocal features^31^. Nearly all studies of vocal expression in schizophrenia have employed small feature acoustic sets—on the order of 2–10 features^25^. Thus, it could be the case that larger and more conceptually diverse acoustic feature sets can capture BvA and alogia in ways more limited features sets cannot.

**Fig. 1:**
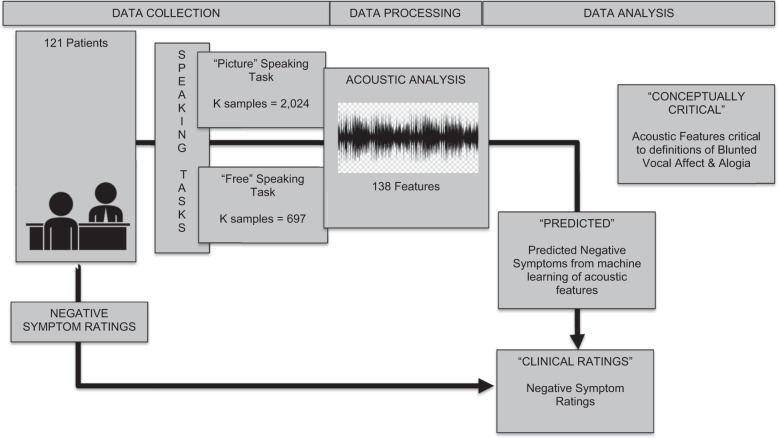
Methods and critical terms used in this study. This figure depicts the data collection, processing and analysis stages of the project. It also defines critical terms used throughout the paper.

The present study applied regularized regression, a machine-learning procedure that can accommodate large feature sets without inherently increasing type 1 errors or overfitting, to a large corpus of speech samples from two archived studies of patients with SMI. Data using conventional “small feature set” analyses (i.e., 2–10 features) of these data have been published elsewhere^18,21,32^. Our aims were to: (1) evaluate whether clinically rated BvA and alogia can be accurately modeled from acoustic features extracted from the natural speech of two distinct speaking tasks, (2) evaluate if model accuracy changes as a function of these separate speaking tasks, (3) evaluate the convergence/divergence of BvA/alogia measured using machine learning versus clinical ratings to demographic characteristics, psychiatric symptoms and cognitive and social functioning, and (4) evaluate the key features from the models. This final step involved “opening the contents of the black box”, as it were, to provide potential insight into how BvA/alogia is rated by clinicians. A visual heuristic of the studies methods and key terms is provided in Fig. 1.

## Results

### Can clinically rated blunted affect and alogia be accurately modeled from vocal expression? (Table 1, Supplementary Tables 1 and 2)

Average accuracy across the 10 analytic-folds for BvA and alogia classifications were 90% and 95% respectively for the training sets. Average accuracy from the test sets was similar (i.e., within 5%) and well above chance (i.e., 50%). For all models, this reflected good hit and excellent correct rejection rates. The model features, and their weights, are included in supplementary form (Supplementary Tables 1 and 2).

### Do these models (and their accuracy) change as a function of speaking task (Supplementary Table 3)?

When vocal expression for the Picture and Free Recall speaking tasks were modeled separately, there was a general improvement in hit rate, even though there were fewer samples available for analysis. Accuracy improvement was particularly notable when modeling alogia from the Picture Task data, where average accuracy reached 99% and 96% in the training and test sets respectively. Predicted scores from the BvA and Alogia models (i.e., ML BvA /alogia) showed high convergence with clinical ratings of BvA and Alogia (i.e., clinically rated BvA/alogia; *r*’s = 0.73 and 0.57 respectively, *p*’s < 0.001). Predicted BvA and Alogia scores were modestly related to each other (*r* = 0.25, *p* < 0.01) as were clinical ratings of BvA and Alogia (*r* = 0.46, *p* < 0.01).

To evaluate whether these models were equivalent, we extended our cross-validation approach by applying the models developed for one task to the other task (see Supplementary Table 3). Adjusted accuracy dropped to near chance levels, suggesting that the models were task-specific. For example, accuracy for predicting alogia from a speech in the picture task dropped from 99% (in the training set) to 50% when applied to the Free Speech task. The range of adjusted accuracy rates ranged from 0.50 to 0.63; all much lower than those seen in Table 1.

**Table 1:** Summary of ML-based analyses, predicting clinical ratings of blunted vocal affect and alogia.

			Training set			Test set		
Criterion	Speaking task	K Neg/Pos cases	Hit rate	Correct rejection	Accuracy	Hit rate	Correct rejection	Accuracy
Blunted vocal affect	All	1204/404	0.74	0.95	0.90	0.65	0.92	0.85
Blunted vocal affect	Picture task	915/317	0.84	0.97	0.94	0.70	0.93	0.87
Blunted vocal affect	Free speech	289/87	0.88	0.99	0.96	0.60	0.93	0.85
Alogia	All	1452/253	0.75	0.98	0.95	0.66	0.96	0.92
Alogia	Picture task	1220/140	0.89	1.00	0.99	0.76	0.99	0.96
Alogia	Free speech	232/113	0.96	0.98	0.97	0.82	0.92	0.89

*ML* machine learning.

These data suggest that model accuracy is enhanced by considering speaking task. For consequent analyses, we employed machine-learning scores from the Picture Task data, as these models showed the highest accuracy in predicting clinical ratings and had more samples available for analysis. For participants completing the Picture Task, 68% (*N* = 39; *K* audio samples = 915) of participants were BvA negative while 32% (*N* = 18; K = 317 audio samples) were BvA positive. 82% (*N* = 39; *K* audio samples = 1220) of participants were alogia-negative while 18% (*N* = 10; *K* audio samples = 140) were alogia-positive.

### Do machine-learning scores converge with demographic, diagnostic, clinical, and functioning variables? (Table 2 and 3)

Neither predicted scores nor clinical ratings significantly differed between men (*n* = 35) and women (*n* = 22; *t*’s < 1.26, *p*’s > 0.21, *d*’s < 0.35). In contrast, predicted and clinically rated alogia were more severe/higher in men than women at a trend level or greater (*t* = 4.41, *p* < 0.01, *d* = 1.20 and *t* = 1.77, *p* = 0.08, *d* = 0.44) respectively. African-Americans (*n* = 30) and Caucasian (*n* = 27) participants did not significantly differ in predicted scores or clinical ratings (*t*’s < 1.36, *p*’s > 0.18, *d*’s < 0.36). Age was not significantly correlated with predicted scores (absolute value of *r*’s < 0.14, *p*’s > 0.31) or clinical ratings (absolute value of *r’*s < 0.25, *p*’s > 0.07).

Bivariate correlational analysis (Table 2; see Supplementary Table 4 for correlations by task) suggested that predicted scores were not significantly related to non-negative psychiatric symptoms. Importantly, they were not significantly associated with negative affect, hostility/aggressiveness, positive or bizarre behavior; all of which are symptoms associated with secondary negative symptoms^4,33^. Clinical ratings of BvA were associated with more severe positive symptoms and hallucinations. With respect to functioning, more severe predicted BvA was significantly associated with poorer cognitive performance and social functioning. More severe predicted alogia was associated with poorer social functioning. These results were supported using linear regressions (Table 3), where the contributions of predicted scores and clinical ratings to cognitive and social functioning were essentially redundant. Neither contributed significantly to functioning once the other’s variance was accounted for.

**Table 2:** Correlations between clinical variables and ML-predicted/clinically rated scores.

	Blunted vocal affect		Alogia	
	Clinical ratings	Predicted scores	Clinical ratings	Predicted scores
*Global psychiatric symptoms*				
BPRS: Agitation	−0.09	−0.22	0.02	0.15
BPRS: Positive	0.28*	0.06	0.14	−0.02
BPRS: Negative	0.82*	0.63*	0.46*	0.20
BPRS: Affect	−0.11	−0.13	−0.06	0.06
*Schizophrenia-spectrum symptoms*				
SAPS: Hallucinations	0.37*	0.19	0.13	−0.05
SAPS: Delusions	0.27	0.26	0.27	0.05
SAPS Bizarre behavior	0.07	−0.02	0.18	0.03
SAPS: Thought disorder	−0.13	−0.12	0.17	0.19
SANS: Blunted affect	0.85*	0.62*	0.43*	0.20
SANS: Alogia	0.46*	0.34*	1.00	0.57*
SANS: Apathy	−0.17	0.10	0.07	0.00
SANS Anhedonia	0.14	0.18	0.19	0.08
*Functioning*				
Cognition	−0.30*	−0.29*	−0.14	0.03
Social functioning	−0.27^+^	−0.28^+^	−0.28^+^	−0.31*

Bivariate correlations between ML “Predicted” scores (from Machine Learning) and Clinically Rated Blunted Vocal Affect and Alogia scores and clinical symptom and functioning variables. ML scores for each audio recording were averaged across participants (total *K* samples = 1745, *n* = 55).

*ML* machine learning.

*p < 0.05; ^+^*p* < 0.10.

**Table 3:** Contributions of ML-predicted versus clinically rated BvA and alogia to cognitive/social functioning, beyond demographics (entered in step 1).

	DV: Cognitive functioning			DV: Social functioning				
	∆*R*^2^	∆*F*	*B* (se)	∆*R*^2^			∆*F*	*B* (se)
*Symptom of interest: blunted vocal affect (BvA)*								
Unique contribution of Clin Rat BvA								
Step 2: Predicted measure Only	0.08	4.13*	−0.53 (0.23)	0.11			4.62*	−0.66 (0.31)*
Step 3: Clin Rat measure only	0.07	0.47	−0.35 (0.33)	0.00			0.13	−0.05 (0.15)
Unique contribution of ML BvA								
Step 2: Clin Rat measure only	0.07	4.65*	−0.18 (0.08)*	0.07			3.15^+^	−0.18 (0.10)^+^
Step 3: Predicted measure only	0.02	1.14	−0.35 (0.33)	0.04			1.58	−0.55 (0.44)
*Symptom of interest: Alogia*								
Unique contribution of Clin Rat Alogia								
Step 2: Predicted measure only	0.00	0.02	−0.03 (0.018)	0.10*			4.88*	−0.46 (0.21)*
Step 3: Clin Rat measure only	0.02	0.90	−0.17 (0.18)	0.01			0.55	−0.18 (0.24)
Unique contribution of ML alogia								
Step 2: Clin Rat measure Only	0.01	0.74	−0.13 (0.14)	0.09*			4.11*	−0.36 (0.18)
Step 3: Predicted measure Only	0.01	0.18	0.09 (0.22)	0.03			1.32	−0.32 (0.28)

Note: Step 1 demographics *R*^2^ = 0.19; Step 1 demographics *R*^2^ = 0.01.

*ML* machine learning, *BvA* blunted vocal affect, *Clin Rat* clinical rating, *se* standard error.

*p < 0.05.

Predicted scores were next compared as a function of DSM diagnosis (Supplementary Fig. 1). The “Other SMI” group was excluded from statistical analysis, as it included relatively few participants. There were statistically significant group differences for machine-learning and clinically rated BvA (*F*’s = 4.30 and 4.33, *p’*s = 0.04), but not alogia (*F*’s < 2.14, *p*’s > 0.15). The schizophrenia group showed medium effect size differences in machine learning-based BvA compared to the mania (*d* = 0.50) and depression (*d* = 0.79) groups. The mania and depression groups were relatively similar (*d* = 0.26).

### What are the key model features? (Table 4 and Supplementary Table 1)

Stability selection analysis yielded two and three critical features for predicting clinically rated BvA and alogia respectively (Table 4). These features are notable for two reasons. First, these features do not appear central to their operational definitions. Thus, the features identified as most “stable” are not necessarily those that are most conceptually relevant to clinically rated negative symptoms. Second, there was an overlapping feature between the models: “F2frequency_sma3nz_amean”. This feature is primarily related to vowel shaping, and while not entirely unrelated to BvA or alogia, it is not a feature with substantial conceptual overlap. It is noteworthy that conceptually critical features are, in some cases, highly inter-correlated (see next section for elaboration), and thus were potentially unstable across iterations of the stability selection analysis. Nonetheless, it is unexpected that the selected features were so distally related to the conceptual definitions of BvA and alogia.

**Table 4:** The most stable features for predicting BvA and alogia from vocal acoustics.

Feature name	How feature is computed	What feature means
*Alogia*		
Unvoiced Segment Length: SD (StddevUnvoicedSegmentLength)	Standard deviation of unvoiced segments length	Captures the variability in pause length. This is potentially related to articulation rate and speech production, and conceptually critical to alogia.
*Blunted affect*		
Mel-Frequency-Capstral-Coefficients – 2: SD (mfcc2_sma3_stddevNorm)	Computed as a spectrum of transformed frequency values over time	Captures variability in the global signature of the signal spectrum over time, based on a short-term frequency representation based on a nonlinear mel scale of frequency. It broadly reflects global changes in the vocal tract and is critical for speech recognition in humans and in automated systems. The MFCC2 reflects finer spectral details than MFCC1.
Harmonic Difference: H1 – A3 (logRelF0-H1-A3_sma3nz_amean)	Mean ratio of energy of the first F0 harmonic (H1) to the energy of the highest harmonic in the third formant range (A3)	Ratio of energy of the first F0 harmonic to the third F0 harmonic - generated from the vocal folds as opposed to the vocal tracts. A measure of “spectral tilt” (i.e., tendency for lower frequencies to have less volume), and associated with breathy voice in men, and lack of “creaky voice”
*Both blunted vocal affect and alogia*		
Second Formant: M (F2frequency_sma3nz_amean)	Average of formant 2 frequency values	Captures spectral shaping of vocal signal, computed as the average frequency from vowel shaping. The second formant typically reflects tongue body movement from front to back.

Acoustic features determined to be most stable using stability selection.

*BvA* blunted vocal affect.

The relative unimportance of “conceptually critical” features in the models was corroborated with additional analyses. First, we inspected the top features in the models (Supplementary Tables 1 and 2), and those with relatively modest to high feature weights (i.e., values exceeding 1.0), creating a “sparse matrix”. A minority of features in the alogia model were directly tied to speech production: only three of 17 (i.e., “utterance_number”, “silence_percent”, “StddevUnvoicedSegmentLength”). Similarly, a minority of features in the BvA were directly (and exclusively) tied to fundamental frequency or intensity values—approximately 11 of 40. In contrast, features related to the Mel-Frequency cepstrum (MFCC), spectral, and formant frequency (i.e., F1, F2, and F3 values) were well represented in both models, with ~12 of 17 of the top features in the sparse matrix predicting alogia, and ~24 of 40 in the sparse matrix predicting BvA. Second, the correlational analysis suggested that our conceptually critical features were often not highly associated with machine learning or clinical rating measures (Supplementary Tables 1 and 2). Of the 12 potential correlations computed between predicted scores, clinical ratings and the four conceptually critical measures defined in the “acoustic analysis” section above, only three were statistically significant and only one was in the expected direction (Supplementary Table 5). Decreased intonation was associated with more severe machine-learning alogia (*r* = −0.55, *p* < 0.01), and increased pause times were significantly associated with machine learning and clinically rated BvA (*r’*s 0.30 and 0.29 respectively, *p*’s < 0.05), but not with alogia.

### Follow-up analyses: how important are conceptually critical features in explaining functioning? (Table 5)

Given that alogia is generally defined in terms of long pauses and few utterances, and BvA is generally defined in terms of relatively monotone speech with respect to intonation and emphasis, we examined whether acoustic features more directly tapping these abilities explained variance in cognitive and social dysfunction beyond the predicted scores and clinical ratings. For regression analysis, we examined demographics (step 1), predicted scores and clinical ratings (step 2), and conceptually critical features (step 3) in their prediction of cognitive functioning (model 1) and social functioning (model 2). Pause mean time and Utterance numbers were highly redundant (*r* = −0.90), so we omitted the latter from our regression models. Both Pause Mean and Emphasis made significant contributions in predicting cognitive functioning. This suggests that Pause Mean and Emphasis, conceptually critical components to alogia and BvA respectively, explain aspects of cognitive functioning missed by predicted scores and clinical ratings.

## Discussion

The present study examined whether clinically rated BvA and alogia could be modeled using acoustic features from relatively brief audio recordings in a transdiagnostic outpatient SMI sample. This study extended prior literature by using a large acoustic feature set and a machine-learning-based procedure that could accommodate it. There were four notable findings. First, we were able to achieve relatively high accuracy for predicting BvA and alogia. Second, this accuracy improved when we took speaking task into consideration, suggesting that model solutions are not ubiquitous across speaking tasks and recording samples. Third, there were no obvious biases with respect to demographic characteristics in our prediction model that were not also present in clinical ratings (see below for elaboration). Fourth, predicted scores were essentially redundant with clinical ratings in explaining demographic, clinical, cognitive, and social functioning variables. Finally, the acoustic features most stable for predicting clinical ratings were not necessarily those most conceptually relevant. These additional “conceptual-based” features explained unique variance in cognitive functioning, raising the possibility that clinicians are missing critical aspects of alogia, at least as operationally defined, when making their ratings.

From a pragmatic perspective, the present study reflects an important “proof of concept” for digitally phenotyping key negative symptoms from brief behavioral samples^10,13^. Clinical ratings can be expensive in terms of time, staff, and space resources and generally require active and in vivo patient participation. The promise of efficient and accurate digital phenotyping of audio signal can significantly reduce these burdens, and offer potential remote assessment using archived, telephone, and other samples. Moreover, the use of ratio-level data can improve sensitivity in detecting subtle changes in symptoms for clinical trials of psychosocial and pharmacological interventions, monitoring treatment side-effects, and designing biofeedback interventions^34^. Highly sensitive measures of negative symptoms can also be potentially important for understanding their environmental antecedents. It could be the case that, for example, a particular individual tends to show BvA and alogia primarily with respect to positively-, but not negatively valenced emotion^35^, or when their “on-line” cognitive resources are sufficiently taxed^21^. It is well known that negative symptoms are etiologically heterogeneous^4,33^, and sensitive measures able to track their severity as individuals navigate their daily environment could be essential for understanding, measuring, and addressing the various primary and secondary causes of negative symptoms.

In terms of optimizing ML solutions for understanding negative symptoms using behavioral samples, it is important to consider speaking task and individual differences. While good accuracy was obtained in the present study regardless of speaking task, model accuracy did improve when speaking task was considered. The tasks examined in this study were relatively similar to each other in that they were brief, were conducted in a laboratory setting and involved interacting with a relative stranger. Hence, it is not clear whether the models derived in this study are of any use for predicting speech procured from other settings/contexts. Model accuracy in this study did not notably differ as a function of gender or ethnicity beyond those differences observed with clinical ratings. Importantly, the participants in this study were sampled from a constrained geographic catchment region and reflect a limited representation of the world’s diverse speaking styles. Moreover, gender differences were observed in both the clinical ratings and ML measures of BvA. While clinically rated negative symptoms are commonly reported as being more severe in men than women^36^, and in African Americans versus Caucasians^37^, it is as yet unclear whether this reflects a genuine phenotypic expression, a cultural bias of clinicians, or bias in the operational definitions of negative symptoms. Regardless, a machine-learning model built on biased criteria will show similar biases. Examples of this in computerized programs analyzing objective behavior are increasingly becoming a concern^38,39^.

The present study offers a unique insight into the acoustic features that clinicians consider critical for evaluating BvA and alogia. Features related to the MFCC, spectral, and formant frequency (i.e., F1, F2, and F3 values) were particularly important, as they represented at least half of the top features in models predicting alogia and three-quarters of the top in models predicting BvA. These features have not been typically examined in the context of SMI, as they are not captured by the VOXCOM or CANS systems (refs. ^40,41^; but see^12,42^ for an exception). Collectively, these features concern the spectral quality and richness or speech, reflecting the involvement of a much broader vocal system than those typically involved in psychopathology research, e.g., “pitch” and “volume”. These “spectral” measures involve coordination between vocal tracts, folds and involve the shaping of sounds with mouth and tongue^43^. The MFCC values have become particularly important for speech and music recognition systems, and figure prominently in machine learning-based applications of acoustic features more generally^44,45^. Applications for understanding SMI are relatively limited, though links between reduced MFCC and clinically rated depression in adults (e.g., with 80% classification accuracy^46^) and adolescents (e.g., with 61.1% classification accuracy^47^) have been observed. Moreover, Compton et al.^12,42^ have demonstrated statistically significant relationships between formant frequencies and clinically rated negative symptoms (e.g., *r* = −0.45^12^). In short, clinicians appear to be intuitively evaluating a much broader feature set than is included in most prior studies.

However, it is not entirely clear that clinicians are accurately capturing the most essential acoustic features of patient speech when evaluating BvA and alogia. It is unexpected that pause length and utterance number didn’t figure more prominently in models predicting alogia, and that F0 and intensity variability (i.e., intonation and emphasis) didn’t figure more prominently in models predicting BvA. There are two potential explanations for this. First, it could be that clinicians are correctly ignoring aspects of vocal expression that are included in the operational definitions of BvA and alogia but are actually nonessential to negative symptoms. If true, the operational definitions of BvA and alogia should be updated accordingly. Second, it could be that clinicians aren’t accurately evaluating features that are critically relevant to BvA and alogia. Alpert and colleagues^48^ have proposed that clinical ratings of vocal deficits are conflated by perceptions of global impressions of patient behavior rather than precise evaluations of relevant behavioral channels; experimental and correlational support for this claim exists^11,19,48–51^. While beyond the present study to resolve, a major challenge in digital phenotyping and modeling of clinical symptoms more generally involves defining the “ground truth” criteria. Should models be built to predict facets of psychopathology defined based on conceptual models, based on clinician ratings, or based on other variables, such as cognitive, social or other dysfunctions more generally? Importantly, a burgeoning area of medicine includes developing “models of models”^52^, and this may allow integration of models built on different criteria. Nonetheless, deciding on the optimal criterion for a model, and how it should reflect clinical ratings, dysfunction or theory, is critical to the field of computational psychiatry.

It is a bit surprising that measures of alogia (particularly predicted from machine learning) weren’t more related to cognitive functioning. In at least some prior studies, measures of speech production have been associated with measures of attention, working memory, and concentration^21,51^, and experimental manipulation of the cognitive load has caused exaggerated pause times in patients with SMI^21^. Some have proposed that cognitive deficits reflect a potential cause of alogia^2^, and may differentiate schizophrenia from mania-psychosis^53^. In the present study, average pause times explained 15% of the variance in cognitive functioning beyond the negligible contribution made by clinical ratings and predicted scores (Table 5). This supports the notion that there is something important in the operational definitions of alogia that is missed by clinical ratings. The lack of replication in this study could also reflect context, as the speech tasks were not particularly taxing in cognitive resources—at least, in terms of overall speech production. It could be that patient’s speech was informationally sparser, for example, characterized by more “filler” words (e.g., “uhh”, “umm”), more repetition, and more automated or cliched speech. This highlights a potential limitation of relying solely on acoustic analysis, in that other aspects of vocal communication are not considered. This is being addressed in other lines of research^54,55^.

**Table 5:** Contributions of Conceptually Critical Features for predicting cognitive/social functioning, beyond demographics (entered in step 1).

Conceptually critical features:	DV: Cognitive functioning			DV: Social functioning		
	∆*R*^2^	∆*F*	*B* (se)	∆*R*^2^	∆*F*	*B* (se)
*Symptom of Interest: blunted vocal affect*						
Intonation	0.01	0.78	−0.21 (0.21)	0.02	1.17	0.30 (0.28)
Emphasis	0.06	4.52*	−0.40 (0.19)*	0.00	0.12	−0.08 (0.24)
*Symptom of Interest: Alogia*						
Pause mean	0.13	9.59*	−0.54 (0.17)*	0.01	0.25	−0.11 (0.22)

Relative contributions of Conceptually Critical Features (Step 3) beyond that of Predicted and Clinical Symptom Rating measures (Step 2) and demographics (Step 1) for predicting cognitive and social functioning (Dependent variables; DV). Note: Step 1 demographics *R*^2^ = 0.19; Step 1 demographics *R*^2^ = 0.12; Step 1 demographics *R*^2^ = 0.27; Step 1 demographics *R*^2^ = 0.12.

*se* standard error, *DV* dependent variable.

*p < 0.05.

Some limitations warrant mention. First, we were unable to meaningfully evaluate the role of primary versus secondary negative symptoms, or the degree to which these symptoms were enduring. It is possible that alogia and BvA secondary to depression, medication side effects, or anxiety, for example, differ in their vocal sequelae and in their consequent predictive modeling. There were no significant correlations between machine learning-based scores and psychiatric symptoms other than negative symptoms. Nonetheless, this is an important area of future research. Second, the speech tasks were relatively constrained. This issue was compounded by the fact that sample sizes were relatively low for the free speech task. Future studies should involve a greater breadth of speaking tasks, to include those that are more natural (spontaneous conversation) to those that are even more constrained (e.g., memory tasks) in terms of task demands, and ensure that there are adequate samples for analysis. Cross-validation involving novel data is important for generalization, and also for addressing potential overfitting in the training models. The latter is something we could not optimally address in the present study using our cross-validation strategy. Third, extreme levels of negative symptom severity were not particularly well represented in our sample. Although extreme BvA and alogia are not particularly common within the outpatient populations, future modeling should ensure a good representation of patients with extreme levels of alogia (either present or absent). Finally, we did not control or account for the effects of medication.

## Methods

### Participants (Supplementary Table 6)

Participants (*N* = 121 total; 57 in Study 1 and 64 in Study 2) were stable outpatients meeting United States federal definitions of SMI per the ADAMHA Reorganization Act with a depressive, psychosis or bipolar spectrum diagnosis and current and severe functional impairment. All were receiving treatment for SMI from a multi-disciplinary team and were living in a group home facility. The sample was ~61% male/39% female and 51% Caucasian/48% African-American. The average age was 41.88 (standard deviation = 10.95; range = 18–63). Approximately two-thirds of the sample met criteria for schizophrenia (*n* = 76), with the remainder meeting criteria for major depressive disorder (*n* = 18), bipolar disorder (*n* = 20), or other SMI disorders (e.g., psychosis not otherwise specified; *n* = 7). Participants were free from major medical or other neurological disorders that would be expected to impair compliance with the research protocol. Participants did not meet the criteria for DSM-IV substance dependence within the last year, as indicated by a clinically relevant AUDIT/DUDIT score^56,57^. The reader is referred elsewhere for further details about the participants (Study 1^32^; Study 2^21^; a collective reanalysis of this, and other data^18^). All data were collected as part of studies approved by the Louisiana State University Institutional Review Board. Participants offered written informed consent prior to the study, but were not asked whether their raw data (e.g., audio recordings) could be made public. For this reason, the raw data are not available to the public. The processed, de-identified datasets generated analyzed for this current study are available from the corresponding author on reasonable request

### Measures

#### Clinical measures

Structured clinical interviews^58^ were conducted by doctoral students under the supervision of a licensed psychologist (AS Cohen). Psychiatric symptoms were measured using the Expanded Brief Psychiatric Rating Scale (BPRS^59^) and the Scales for the Assessment of Positive and Negative Symptoms (SAPS & SANS^60,61^). Diagnoses and symptom ratings reflected consensus from the research team. For the BPRS, we used scores from a factor solution^62^ with some minor modifications to attain acceptable internal consistency (>0.70). For the SANS, we used the BvA and global alogia ratings as a criterion for the machine-learning modeling—those most relevant to our acoustic features. To evaluate the convergent/divergent validity of our models, we used the global scores from the SAPS/SANS. Symptom data were missing for 407 of the audio samples.

#### Cognitive and social functioning

Cognitive functioning was measured using the Repeatable Battery for the Assessment of Neuropsychological Status global cognitive index score (RBANS^63^). Social functioning was measured using the Social Functioning Scale: Total score (SFS^64^). These measures were available for Study 1 data only.

#### Speaking tasks

Participants were audio-recorded during two separate tasks. The first involved discussing reactions to visual pictures displayed on a computer screen for 20 s. This “Picture Task” was administered in Study 1, and involved a total of 40 positive, negative- and neutral-valenced images from the International Affective Picture System (IAPS)^65^ shown to patients across one of two testing sessions (20 pictures each session; with sessions scheduled a week apart). Participants were asked to discuss their thoughts and feelings about the picture. The second task involved patients providing “Free Recall” speech describing their daily routines, hobbies and/or living situations, and autobiographical memories (Studies 1 and 2) for 60 s each. While these tasks weren’t designed as part of an a priori experimental manipulation, they do systematically differ in length, constraints on speaking topic, and personal relevance. The administration was standardized such that, for all tasks, instructions, and stimuli presentation (e.g., IAPS slides) were automated on a computer, and participants were encouraged to speak as much as possible. Research assistants were present in the room, and read instructions to participants, but were not allowed to speak while the participant was being recorded.

### Acoustic analysis

Acoustic analysis was conducted using two separate, conceptually different, software programs. The first was designed to capture relatively global features conceptually relevant in psychiatric symptoms and reflects an iteration of the VOXCOM system developed by Murray Alpert^40^. The second captures more basic, psychophysically complex features relevant to affective science more generally. The first was the Computerized assessment of Affect from Natural Speech (CANS)^66,67^. Digital audio files were organized into “frames” for analysis (i.e., 100 per second). During each frame, basic speech properties are quantified, including fundamental frequency (i.e., frequency or “pitch”) and intensity (i.e., volume) and summarized within vocal utterances (defined as silence bounded by 150+ milliseconds). Support for the CANS comes from over a dozen studies from our lab, including psychometric evaluation in 1350 nonpsychiatric adults^67^ and 309 patients with SMI^18^. The CANS feature set includes 68 distinct acoustic features related to speech production (e.g., number of utterances, average pause length) and speech variability (e.g., intonation, emphasis). The second program involved the Extended Geneva Minimalist Acoustic Parameter Set (GeMAPS)^31^. GeMAPS was derived using machine learning-based feature reduction procedures on a large feature set as part of the INTERSPEECH competitions from 2009 to 2013^68^. GeMAPS contains 88 distinct features. Validity for this feature set, for predicting emotional expressive states in demographically diverse clinical and nonclinical samples, can be found elsewhere (e.g.^69^). Recordings that contained fewer than three utterances were excluded from analyses.

As part of exploratory analyses, we selected four features from our CANS analysis deemed “conceptually critical” to the operational definitions of BvA (i.e., intonation: computed as the average of the standard deviation of fundamental frequency values computed within each utterance; emphasis: computed as the average of the standard deviation of intensity/volume values computed within each utterance) and alogia (i.e., mean pause time: average length of pauses in milliseconds; the number of utterances: number of consecutively voiced frames bounded on either side by silence). These are by no means comprehensive, nonetheless, they reflect “face valid” proxies of their respective constructs. These features have been extensively examined in psychiatric and nonpsychiatric populations, and reflect key features identified in principal components analysis of nonpsychiatric and psychiatric samples^11,18,40,67^.

### Analyses: aims and statistical approaches

Our analyses addressed four aims. First, we were interested in evaluating whether clinical ratings could be accurately modeled from acoustic features. We hypothesized that good accuracy would be achieved (i.e., exceeding 80%). Second, we evaluated whether model accuracy changed as a function of the speaking task. Third, we used the models derived from the first two aims to compute machine learning-based “predicted” scores for each vocal sample. These scores were then examined in their convergence with demographic (i.e., age, gender, ethnicity), diagnostic (i.e., DSM IV-TR diagnosis), clinical symptom (BPRS and SANS/SANS factor/global ratings) and cognitive (i.e., RBANS total scores) and social (i.e., SFS total scores) functioning variables. We employed linear regressions comparing the relative contributions of predicted versus clinician-rated symptoms in predicting social and cognitive functioning. Regressions, as opposed to multi-level modeling, were necessary due to the dependent variables being “level 2” variables (i.e., reflecting data that are invariant across sessions within a participant). For these analyses, scores were averaged within participants. Correlations and group comparisons were included for informative purposes. We hypothesized that predicted scores and clinician ratings would explain similar variance in cognitive and social functioning, given that the ML models are built to approximate, as closely as possible, the clinical ratings. Fourth, we identified and qualitatively evaluated the individual acoustic features associated with each model. This was done by inspecting the model weights, correlations, and by using stability selection (see next section). Generally, we expected that predicted scores would be highly related to acoustic features deemed conceptually critical to their operational definitions. A limited set of these was identified for alogia (i.e., mean pause times, the total number of utterances) and BvA (i.e., intonation, emphasis). All data were normalized and trimmed (i.e., “Winsorized” at 3.5/−3.5) before being analyzed.

#### Machine learning

We employed Lasso regularized regression, with 10-fold cross-validation^70^. Each case in the dataset was one of ten groups such that the ratio of positive and negative cases in each group was the same. Then, a test set was formed by selecting one of these groups, and a training set was formed by combining the remaining nine groups. A model was fit to the training set and evaluated on the test set. This was repeated so that each of the 10 groups was used as the test set. We report hit rate and correct rejection values that have been averaged over these 10 folds. We report accuracy as the sum of the hit rate and correct rejection rate divided by 2 so that 0.5 corresponds to random performance. Feature selection was informed by stability selection^71^, a subsampling procedure that resembles bootstrapping^72^. Additional information is available in Supplementary Note 1.

For model building purposes, we defined positive and negative cases of BvA/alogia based on SANS ratings of “moderate” or greater, and “absent” severity of symptoms respectively. Cases where SANS ratings were “questionable” or “mild” were excluded from model building. After satisfactory models were established, they were then used to compute individual “predicted” scores for all data (i.e., including, “questionable” and “mild” cases). Given that our model was based on binary classification, this helped to remove potentially ambiguous cases when building our model and helped define the extreme “ends” of the continuum when applied to individual scores (i.e., with zero reflecting “absent” and one reflecting “moderate and above). Our use of a binary criterion is not meant to imply that the symptom is binary in nature; modeling allows a “degree of fit” score that is continuous in nature. Using these criteria, 64% (*n* = 77; *k* audio samples = 1671) of participants were BvA negative while 36% (*n* = 44; *k* = 825 audio samples) were BvA positive. 70% (*n* = 85; *k* audio samples = 1916) of participants were alogia-negative while 30% (*n* = 36; *k* audio samples = 580) were BvA alogia-positive.

### Reporting summary

Further information on experimental design is available in the Nature Research Reporting Summary linked to this article.

## Supplementary information

Supplemental Materials

Reporting Summary

## Data Availability

Supplemental findings supporting this study are available on request from the corresponding author [ASC]. The data are not publicly available due to Institutional Review Board restrictions—since the participants did not consent to their data being publicly available.

## Code availability

Code used for data processing and analysis is available on request from the corresponding author [ASC]
