# Supplementary material for: Using machine learning of computerized vocal expression to measure blunted vocal affect and alogia
Source: NPJ Schizophr. 2020 Sep 25;6:26. doi: 10.1038/s41537-020-00115-2 (PMC7519104; doi:10.1038/s41537-020-00115-2)
Supplement: Supplementary file 1 — Supplemental Materials [file 41537_2020_115_MOESM1_ESM.pdf]

Supplementary Material

Supplementary Note 1

Notes on the use of Lasso for this project

Lasso regularized regression has two objectives. The first, as in all models, is to minimize the difference between the true and predicted values of the dependent variable. The second objective is to minimize some function of the model coefficients themselves. In the case of LASSO, the function is the sum of the absolute value of the model coefficients. This term is minimized when all coefficients are set to zero, but such a model completely disregards the data and so will fail to accurately predict the dependent variable. The right balance of regularization and predictive accuracy is different for each dataset, and so we estimate this balance through cross-validation to obtain a model with many—but not all—weights set to zero. By explicitly selecting highly informative features and constraining model degrees of freedom, LASSO can produce models that generalize well to new data.

The strength of the LASSO regularization penalty was tuned to each training set with cross-validation. This is nested within the procedure described above, and importantly never includes examples included in the test set. The penalty is refit to each training set so that the definition of the penalty is completely uninfluenced by aspects of the current test set.

Each round of cross validation will produce a different model, which means potentially a different selection of features. After determining that ML models can generalize to untrained cases, a follow up analysis of important features was carried out based on models fit to all cases and employing stability selection (Meinshausen & Bühlmann, 2010). Stability selection is a subsampling procedure that resembles bootstrapping. By training on thousands of random subsets of cases, we can estimate the probability of each feature being included in the model given different amounts of regularization. Sets of features identified through stability selection are reliably important over many sets of cases (Shah & Samworth, 2013). The inclusion threshold is set to control the familywise error rate.

Meinshausen, N. & Bühlmann, P. Stability selection. J. R. Stat. Soc. Ser. B Stat. Methodol. (2010). doi:10.1111/j.1467-9868.2010.00740.x

Shah, R. D. & Samworth, R. J. Variable selection with error control: Another look at stability selection. J. R. Stat. Soc. Ser. B Stat. Methodol. (2013). doi:10.1111/j.1467-9868.2011.01034.x

Supplementary Figure 1. Machine learning Based alogia (top panel) and blunted vocal affect plotted (y-axis) as a function of diagnostic group.

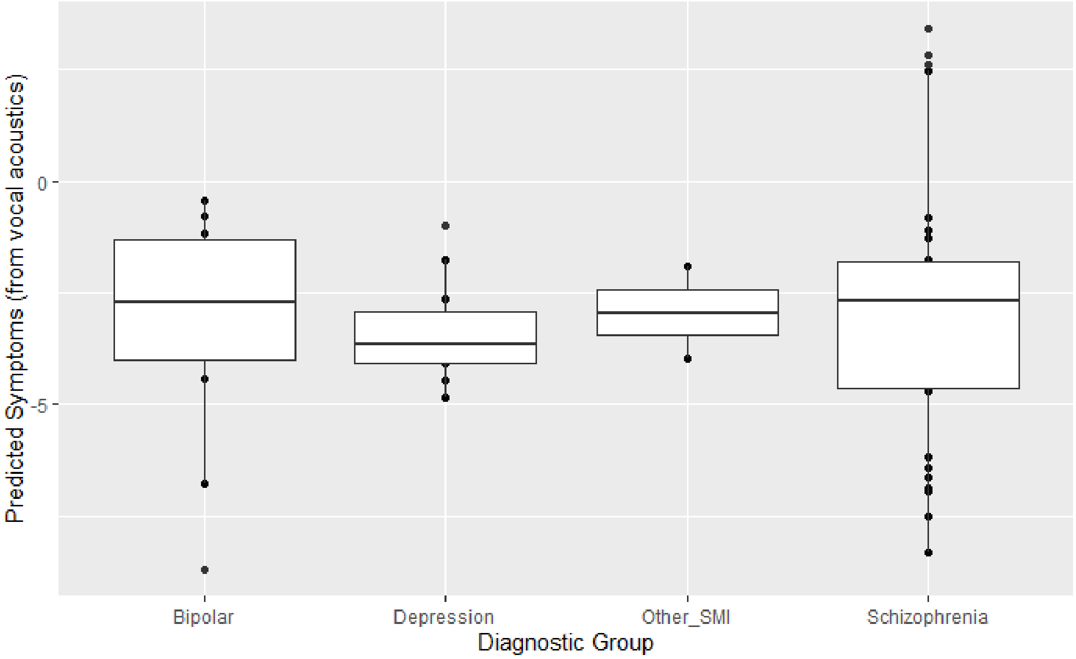

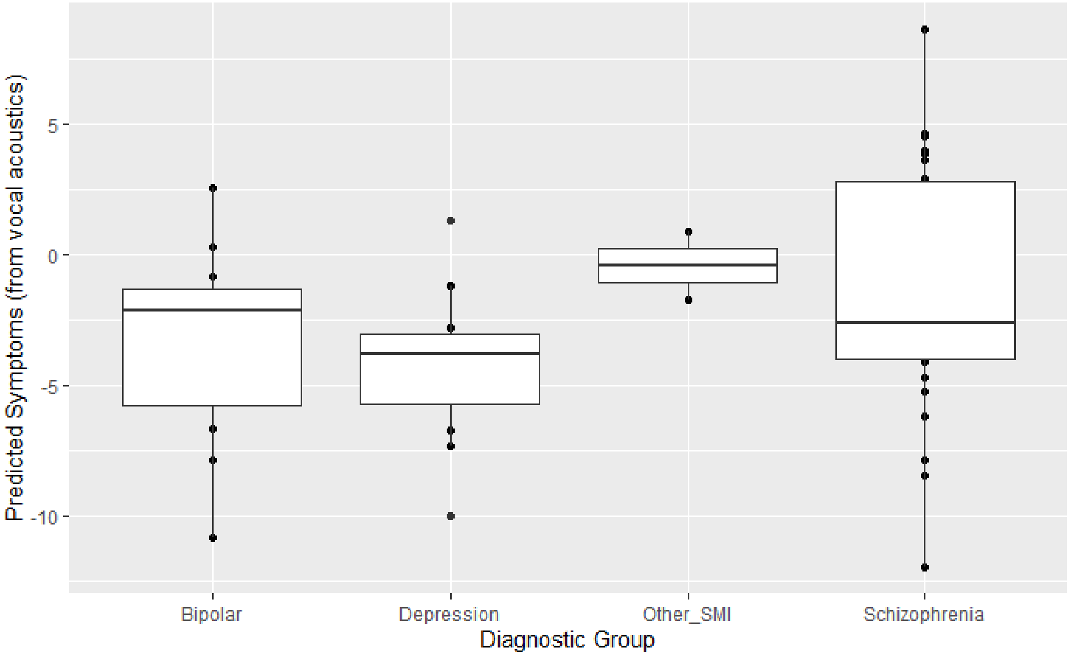

Note: Center lines reflect mean predicted symptom values. Bounds of box and whiskers reflect 95% confidence intervals around means. Black dots reflect individual data points.

Supplementary Table 1. Sparse matrix of model features and relevant weights predicting alogia

Feature	Weight	Correlation with ML BvA
mfcc3_sma3_stddevNorm	19.62	0.13
(Intercept)	-5.13	NA
slopeV0-500_sma3nz_amean	3.77	-0.09
background_mean	3.55	0.22
utterance_number	3.02	0.15
silence_percent	2.91	0.03
mfcc2_sma3_stddevNorm	-2.83	0.07
F2frequency_sma3nz_amean	-2.81	-0.65
slopeUV500-1500_sma3nz_amean	2.58	0.00
alphaRatioV_sma3nz_stddevNorm	2.04	0.05
F3frequency_sma3nz_amean	2.04	-0.61
StddevUnvoicedSegmentLength	-1.89	-0.21
slopeUV0-500_sma3nz_amean	1.82	0.04
F1bandwidth_sma3nz_amean	1.74	0.23
mfcc3V_sma3nz_amean	1.69	-0.05
f0_mean	-1.47	-0.62
spectralFlux_sma3_stddevNorm	-1.28	-0.24
F2bandwidth_sma3nz_amean	1.04	0.35
F2amplitudeLogRelF0_sma3nz_amean	-0.99	0.15
mfcc4_sma3_amean	0.79	0.09
F3frequency_sma3nz_stddevNorm	-0.76	0.03
loudness_sma3_meanRisingSlope	0.75	0.29
F1frequency_sma3nz_stddevNorm	0.74	0.28
f2_range_sd	-0.71	-0.41
logRelF0-H1-H2_sma3nz_amean	-0.71	-0.34
mfcc2V_sma3nz_stddevNorm	0.70	0.04
mfcc3V_sma3nz_stddevNorm	-0.69	0.01
logRelF0-H1-H2_sma3nz_stddevNorm	0.68	0.06
f0_range_mean	-0.66	-0.50
F0semitoneFrom27.5Hz_sma3nz_meanFallingSlope	0.64	-0.30
pause_median	-0.62	-0.12
f1_sd_local	-0.56	-0.12
F2frequency_sma3nz_stddevNorm	0.56	0.28
intensity_pert	-0.54	-0.39
f1_mean	0.49	0.32

hammarbergIndexV_sma3nz_amean	-0.47	-0.26
mfcc2V_sma3nz_amean	-0.45	-0.19
number_500_pauses	0.45	0.00
spectralFluxV_sma3nz_stddevNorm	0.44	-0.32
utterance_sd	0.42	-0.14
mfcc4_sma3_stddevNorm	-0.41	-0.05
f2_sd_local	0.40	-0.34
intensity_mean	-0.40	0.05
logRelF0-H1-A3_sma3nz_amean	-0.39	-0.05
intensity_sd_local	0.38	-0.07
number_1000_pauses	-0.38	-0.11
MeanVoicedSegmentLengthSec	-0.35	0.13
loudness_sma3_stddevFallingSlope	0.33	0.26
f0_sd_local	-0.31	-0.51
intensity_sd_global	0.31	0.48
f1_slope_mean	0.30	0.37
f2_slope_sd	-0.27	-0.08
intensity_range_sd	0.25	0.44
hammarbergIndexV_sma3nz_stddevNorm	-0.23	-0.03
f1_range_sd	-0.19	0.01
F3bandwidth_sma3nz_stddevNorm	-0.18	-0.43
shortest_pause	-0.18	-0.26
f0_slope_mean	0.17	0.09
jitterLocal_sma3nz_amean	-0.16	-0.26
loudnessPeaksPerSec	0.16	0.03
logRelF0-H1-A3_sma3nz_stddevNorm	-0.15	-0.29
f1_slope_sd	0.14	-0.05
f2_slope_mean	0.13	-0.06
F1bandwidth_sma3nz_stddevNorm	-0.12	-0.54
f0_sd_global	-0.10	-0.33
F0semitoneFrom27.5Hz_sma3nz_stddevRisingSlope	-0.09	-0.36
F1frequency_sma3nz_amean	-0.09	-0.61
shimmerLocaldB_sma3nz_amean	0.08	-0.19
intensity_slope_mean	0.06	0.16
f1_sd_global	0.05	0.01
F0semitoneFrom27.5Hz_sma3nz_pctlrange0-2	-0.04	-0.39
latence_first	-0.04	-0.10
backround_sd	-0.03	0.30
f2_sd_global	0.02	-0.38
f0_range_sd	-0.01	-0.25
alphaRatioUV_sma3nz_amean	0.00	0.12

alphaRatioV_sma3nz_amean	0.00	0.20
equivalentSoundLevel_dBp	0.00	0.23
f0_pert	0.00	-0.04
f0_slope_sd	0.00	-0.32
F0semitoneFrom27.5Hz_sma3nz_amean	0.00	-0.60
F0semitoneFrom27.5Hz_sma3nz_meanRisingSlope	0.00	-0.43
F0semitoneFrom27.5Hz_sma3nz_percentile20.0	0.00	-0.52
F0semitoneFrom27.5Hz_sma3nz_percentile50.0	0.00	-0.60
F0semitoneFrom27.5Hz_sma3nz_percentile80.0	0.00	-0.64
F0semitoneFrom27.5Hz_sma3nz_stddevFallingSlope	0.00	-0.18
F0semitoneFrom27.5Hz_sma3nz_stddevNorm	0.00	-0.24
f1_range_mean	0.00	-0.11
F1amplitudeLogRelF0_sma3nz_amean	0.00	0.16
F1amplitudeLogRelF0_sma3nz_stddevNorm	0.00	-0.20
f2_mean	0.00	0.06
f2_range_mean	0.00	-0.32
F2amplitudeLogRelF0_sma3nz_stddevNorm	0.00	-0.19
F2bandwidth_sma3nz_stddevNorm	0.00	-0.55
F3amplitudeLogRelF0_sma3nz_amean	0.00	0.15
F3amplitudeLogRelF0_sma3nz_stddevNorm	0.00	-0.20
F3bandwidth_sma3nz_amean	0.00	0.03
hammarbergIndexUV_sma3nz_amean	0.00	-0.11
HNRdBACF_sma3nz_amean	0.00	-0.56
HNRdBACF_sma3nz_stddevNorm	0.00	0.24
intensity_range_mean	0.00	-0.14
intensity_slope_sd	0.00	0.20
jitterLocal_sma3nz_stddevNorm	0.00	-0.41
longest_pause	0.00	-0.10
loudness_sma3_amean	0.00	0.34
loudness_sma3_meanFallingSlope	0.00	0.29
loudness_sma3_pctlrange0-2	0.00	0.36
loudness_sma3_percentile20.0	0.00	0.20
loudness_sma3_percentile50.0	0.00	0.28
loudness_sma3_percentile80.0	0.00	0.35
loudness_sma3_stddevNorm	0.00	0.00
loudness_sma3_stddevRisingSlope	0.00	0.27
MeanUnvoicedSegmentLength	0.00	-0.19
mfcc1_sma3_amean	0.00	0.14
mfcc1_sma3_stddevNorm	0.00	0.05
mfcc1V_sma3nz_amean	0.00	0.18
mfcc1V_sma3nz_stddevNorm	0.00	-0.24

mfcc2_sma3_amean	0.00	-0.12
mfcc3_sma3_amean	0.00	-0.05
mfcc4V_sma3nz_amean	0.00	0.14
mfcc4V_sma3nz_stddevNorm	0.00	0.16
pause_mean	0.00	-0.13
pause_sd	0.00	-0.10
shimmerLocaldB_sma3nz_stddevNorm	0.00	0.28
slopeV0-500_sma3nz_stddevNorm	0.00	0.24
slopeV500-1500_sma3nz_amean	0.00	-0.14
slopeV500-1500_sma3nz_stddevNorm	0.00	0.04
spectralFlux_sma3_amean	0.00	0.37
spectralFluxUV_sma3nz_amean	0.00	0.32
spectralFluxV_sma3nz_amean	0.00	0.38
StddevVoicedSegmentLengthSec	0.00	0.15
utterance_mean	0.00	-0.17
utterance_median	0.00	-0.19
VoicedSegmentsPerSec	0.00	0.12

Supplementary Table 2. Sparse matrix of model features and relevant weights predicting blunted vocal affect

Feature	Weight	Correlation with ML BvA
mfcc3_sma3_stddevNorm	-64.55	-0.48
F2frequency_sma3nz_amean	-8.57	-0.08
F3frequency_sma3nz_amean	5.56	-0.02
silence_percent	4.24	0.37
F0semitoneFrom27.5Hz_sma3nz_percentile50.0	4.15	-0.18
F3amplitudeLogRelF0_sma3nz_stddevNorm	-4.10	0.34
logRelF0-H1-A3_sma3nz_amean	-3.84	0.02
spectralFlux_sma3_amean	3.76	-0.08
F1frequency_sma3nz_amean	3.45	-0.16
spectralFluxUV_sma3nz_amean	-3.28	-0.13
slopeV500-1500_sma3nz_stddevNorm	-2.84	-0.32
mfcc3V_sma3nz_amean	2.79	0.43
mfcc4V_sma3nz_stddevNorm	-2.42	0.03
mfcc4V_sma3nz_amean	1.98	0.20
loudness_sma3_meanRisingSlope	1.91	-0.20
f0_sd_local	1.89	0.03
F0semitoneFrom27.5Hz_sma3nz_percentile80.0	-1.82	-0.26
slopeUV0-500_sma3nz_amean	-1.73	-0.04
HNRdBACF_sma3nz_stddevNorm	-1.61	-0.01
background_mean	-1.59	-0.20
mfcc3V_sma3nz_stddevNorm	1.57	-0.53
longest_pause	-1.55	0.27
alphaRatioV_sma3nz_stddevNorm	-1.53	0.40
f0_range_mean	-1.53	-0.12
slopeV500-1500_sma3nz_amean	-1.46	0.19
F0semitoneFrom27.5Hz_sma3nz_amean	-1.44	-0.23
number_500_pauses	1.40	-0.32
backround_sd	-1.39	-0.28
loudness_sma3_stddevNorm	-1.39	-0.12
utterance_mean	1.38	-0.24
slopeV0-500_sma3nz_amean	1.36	-0.30
mfcc2V_sma3nz_amean	1.3	0.23
slopeUV500-1500_sma3nz_amean	-1.24	0.33
mfcc3_sma3_amean	-1.16	0.20
intensity_pert	-1.15	-0.03
intensity_sd_local	1.09	-0.09

spectralFlux_sma3_stddevNorm	-1.09	-0.21
intensity_range_mean	-1.05	-0.13
mfcc4_sma3_amean	1.03	0.18
mfcc1_sma3_amean	-1.01	-0.45
MeanUnvoicedSegmentLength	-0.99	0.32
F3bandwidth_sma3nz_amean	0.98	-0.11
hammarbergIndexUV_sma3nz_amean	-0.97	-0.11
f0_mean	-0.92	-0.23
StddevUnvoicedSegmentLength	0.92	0.34
mfcc2_sma3_stddevNorm	0.87	-0.21
utterance_median	-0.84	-0.20
utterance_number	0.81	-0.17
hammarbergIndexV_sma3nz_stddevNorm	-0.78	-0.25
logRelF0-H1-A3_sma3nz_stddevNorm	-0.78	-0.18
mfcc1V_sma3nz_amean	0.76	-0.16
loudness_sma3_pctlrange0-2	0.71	-0.17
F2bandwidth_sma3nz_amean	0.70	0.05
F0semitoneFrom27.5Hz_sma3nz_stddevFallingSlope	0.66	-0.07
loudness_sma3_stddevRisingSlope	-0.66	-0.22
f2_mean	0.63	0.34
loudness_sma3_percentile50.0	-0.63	-0.15
intensity_range_sd	-0.62	-0.06
logRelF0-H1-H2_sma3nz_amean	0.62	-0.09
loudness_sma3_stddevFallingSlope	-0.61	-0.22
F0semitoneFrom27.5Hz_sma3nz_meanFallingSlope	-0.59	-0.05
F1bandwidth_sma3nz_stddevNorm	-0.58	-0.17
f0_range_sd	0.56	-0.02
spectralFluxV_sma3nz_amean	-0.53	-0.05
F1amplitudeLogRelF0_sma3nz_amean	-0.52	-0.34
shimmerLocaldB_sma3nz_amean	0.49	0.28
f2_range_mean	-0.46	-0.20
spectralFluxV_sma3nz_stddevNorm	0.43	-0.46
pause_mean	0.42	0.29
shimmerLocaldB_sma3nz_stddevNorm	-0.41	-0.20
MeanVoicedSegmentLengthSec	-0.40	-0.15
VoicedSegmentsPerSec	-0.39	-0.27
F1bandwidth_sma3nz_amean	-0.38	-0.15
f2_sd_local	0.35	-0.18
F3frequency_sma3nz_stddevNorm	0.35	-0.13
StddevVoicedSegmentLengthSec	-0.35	-0.17
f0_sd_global	-0.34	0.14

mfcc2V_sma3nz_stddevNorm	-0.33	-0.15
mfcc2_sma3_amean	0.31	0.11
f1_mean	0.30	0.17
loudness_sma3_amean	-0.30	-0.17
f0_slope_mean	-0.29	0.07
f2_range_sd	-0.29	-0.29
number_1000_pauses	0.28	-0.37
f0_pert	0.26	0.12
loudness_sma3_meanFallingSlope	-0.25	-0.19
loudness_sma3_percentile20.0	-0.25	-0.13
jitterLocal_sma3nz_amean	-0.24	0.18
F2frequency_sma3nz_stddevNorm	0.23	0.13
F0semitoneFrom27.5Hz_sma3nz_pctlrange0-2	-0.20	-0.15
f2_sd_global	0.20	-0.21
f1_range_mean	-0.18	0.00
f1_range_sd	0.18	-0.22
HNRdBACF_sma3nz_amean	-0.18	-0.23
alphaRatioV_sma3nz_amean	-0.17	-0.26
jitterLocal_sma3nz_stddevNorm	-0.17	-0.24
intensity_slope_mean	0.15	-0.10
mfcc4_sma3_stddevNorm	0.15	0.02
f1_slope_sd	-0.13	-0.03
intensity_slope_sd	0.13	-0.01
latence_first	-0.13	0.09
pause_median	-0.13	0.27
f1_sd_global	-0.12	0.06
(Intercept)	-0.11	NA
intensity_mean	0.10	-0.02
intensity_sd_global	0.10	-0.01
F0semitoneFrom27.5Hz_sma3nz_meanRisingSlope	0.09	-0.03
F0semitoneFrom27.5Hz_sma3nz_stddevNorm	0.07	-0.10
f1_slope_mean	-0.07	0.15
F1frequency_sma3nz_stddevNorm	0.07	0.16
F2bandwidth_sma3nz_stddevNorm	-0.07	-0.01
shortest_pause	-0.07	0.25
f1_sd_local	-0.06	0.05
loudnessPeaksPerSec	-0.06	-0.17
equivalentSoundLevel_dBp	-0.04	-0.28
f2_slope_mean	0.04	-0.07
f2_slope_sd	0.04	-0.15
F3bandwidth_sma3nz_stddevNorm	0.04	-0.07

mfcc1V_sma3nz_stddevNorm	-0.04	-0.13
utterance_sd	0.04	-0.27
f0_slope_sd	0.02	-0.12
alphaRatioUV_sma3nz_amean	0.00	0.10
F0semitoneFrom27.5Hz_sma3nz_percentile20.0	0.00	-0.19
F0semitoneFrom27.5Hz_sma3nz_stddevRisingSlope	0.00	-0.17
F1amplitudeLogRelF0_sma3nz_stddevNorm	0.00	0.35
F2amplitudeLogRelF0_sma3nz_amean	0.00	-0.35
F2amplitudeLogRelF0_sma3nz_stddevNorm	0.00	0.36
F3amplitudeLogRelF0_sma3nz_amean	0.00	-0.35
hammarbergIndexV_sma3nz_amean	0.00	0.14
logRelF0-H1-H2_sma3nz_stddevNorm	0.00	-0.06
loudness_sma3_percentile80.0	0.00	-0.17
mfcc1_sma3_stddevNorm	0.00	0.37
pause_sd	0.00	0.26
slopeV0-500_sma3nz_stddevNorm	0.00	-0.26

Supplementary Table 3. Examining generalizability of models, by applying models built on one speaking task to speech from a different task.

**Original Model**	Applied to	Hit Rate	False Alarm	Correct Rejection	Adjusted Accuracy
		**Symptom: Blunted Affect**			
**Picture Task**	Free Speech	0.76	0.60	0.40	0.58
**Free Speech**	Picture Task	0.83	0.56	0.44	0.63
		**Symptom: Alogia**			
**Picture Task**	Free Speech	0.17	0.16	0.84	0.50
**Free Speech**	Picture Task	0.33	0.89	0.72	0.59

Supplementary Table 4. Bivariate correlations between predicted scores for Picture versus Free Recall tasks, and clinical symptom and functioning variables.

	**Blunted Vocal Affect**		**Alogia**	
	Free Recall	Picture	Free Recall	Picture
**Global Psychiatric Symptoms**				
BPRS: Agitation	-0.17	-0.22	-0.12	0.21
BPRS: Positive	0.13	0.06	-0.12	-0.06
BPRS: Negative	0.39*	0.64*	0.10	0.15
BPRS: Affect	-0.14	-0.13	-0.18	0.05
**Schizophrenia-Spectrum Symptoms**				
SAPS: Hallucinations	0.22	0.19	-0.09	-0.08
SAPS: Delusions	0.13	0.26	-0.05	0.00
SAPS Bizarre Behavior	0.15	-0.02	0.07	0.05
SAPS: Thought Disorder	-0.13	-0.12	0.01	0.22
SANS: Blunted Affect	0.41*	0.63*	0.13	0.16
SANS: Blunt Vocal Affect	0.44*	0.73*	0.14	0.20
SANS: Alogia	0.17	0.34	0.31	0.55*
SANS: Apathy	0.07	0.10	-0.09	-0.03
SANS Anhedonia	-0.02	0.18	-0.20	0.05
**Functioning**				
Cognition	-0.35*	-0.30*	-0.03	0.09
Social Functioning	-0.01	-0.28+	-0.17	-0.29*

+p < .10; *****p < .05;

Supplementary Table 5. Bivariate correlations between Conceptually Critical Features (CCF) and Machine Learning (ML) and Clinically Rated (Clin Rat) Blunted Vocal Affect and Alogia scores.

	**Blunted Vocal Affect**		**Alogia**	
**CCF**	Clin Rat	ML	Clin Rat	ML
Pause Mean	0.30*	0.29*	0.10	-0.18
Utterance Number	-0.18	-0.16	0.00	0.24
Intonation	0.05	0.06	-0.17	-0.55*
Emphasis	-0.13	-0.11	0.04	-0.07

Note: ML scores for each audio recording were averaged across participants (total K samples = 1745, n = 55). * = $p < 0.05$; + = $p < 0.10$

Supplementary Table 6. Descriptive statistics for study participants.

	Mean (Standard Deviation)	Potential Range of Scores
BPRS: Agitation [a.]	1.63 (0.83)	1 to 4.5
BPRS: Positive [a.]	2.14 (1.04)	1 to 5.6
BPRS: Negative [a.]	1.91 (1.04)	1 to 5
BPRS: Affect [a.]	2.68 (1.28)	1 to 6
SAPS: Hallucinations [b.]	2.16 (1.6)	1 to 6
SAPS: Delusions [b.]	2.49 (1.44)	1 to 6
SAPS Bizarre Behavior [b.]	1.63 (0.92)	1 to 4
SAPS: Thought Disorder [b.]	1.7 (1.15)	1 to 5
SANS: Blunted Affect [b.]	2.28 (1.37)	1 to 6
SANS: Alogia [b.]	1.56 (0.95)	1 to 4
SANS: Apathy [b.]	3.02 (1.49)	1 to 6
SANS Anhedonia [b.]	2.46 (1.34)	1 to 6
Global Cognition	78.4 (14.05)	50 to 110
Social Functioning	-0.01 (4.63)	-7.77 to 12.19

[a.] Potential range = 1 to 7; [b.] potential range = 1 to 6
